# Visual Outcomes in Malignant Hypertensive Retinopathy Cases: A Clinical and Spectral Domain Optical Coherence Tomography Study

**DOI:** 10.7759/cureus.62945

**Published:** 2024-06-23

**Authors:** Priyadarshini Mishra, Vikas Kanaujia, Divya Kesarwani, Kumudini Sharma, Jayadev Nanda, Prabhaker Mishra

**Affiliations:** 1 Ophthalmology, All India Institute of Medical Sciences, Bhubaneswar, Bhubaneswar, IND; 2 Ophthalmology, Sanjay Gandhi Postgraduate Institute of Medical Sciences, Lucknow, IND; 3 Ophthalmology, Centre For Sight, Patna, IND; 4 Ophthalmology, Hind Institute of Medical Sciences, Lucknow, IND; 5 Biostatistics & Health Informatics, Sanjay Gandhi Postgraduate Institute of Medical Sciences, Lucknow, IND

**Keywords:** cotton wool spots, subretinal fluid, subfoveal choroidal thickness, central macular thickness, sd oct, exudative retinal detachment, hypertensive retinopathy, hypertensive emergency, disc edema, malignant hypertension

## Abstract

Objective

The objective is to correlate visual outcomes in malignant hypertensive retinopathy with changes in systemic causative factors and spectral domain optical coherence tomography (SD OCT) morphologic parameters.

Materials and methods

This is a prospective observational study including patients presenting within two weeks of acute rise of systolic blood pressure (SBP) ≥ 180 mm Hg or diastolic blood pressure (DBP) ≥ 120 mm Hg and with posterior segment involvement in both eyes. Baseline SBP, DBP, mean arterial pressure (MAP), best corrected visual acuity (BCVA), and SD OCT parameters such as central macular thickness (CMT), subfoveal choroidal thickness (SCT), and sub-retinal fluid (SRF) height were measured at presentation and followed monthly up to three months. These variables at baseline and three months were compared and correlated.

Results

Thirty-three patients (66 eyes) having malignant hypertension were included in the study. Diverse clinical presentations noted among patients were optic disc edema, hard exudates in the macula, peripapillary splinter hemorrhage, cotton wool spots, Elschnig spots, exudative retinal detachment, optic neuropathy, and severe exudative retinopathy. SD OCT shows hyperreflective dots and intraretinal fluid with or without SRF. At three months, the mean SBP, DBP, MAP, CMT, SRF, and SCT all decreased significantly from baseline (p<0.001). Changes in SBP, DBP, MAP, and SCT correlated significantly with changes in BCVA (p<0.001).

Conclusion

In malignant hypertensive retinopathy, macular edema with SRF is the major cause of mild-to-moderate decrease BCVA at presentation, but macular ischemia, exudative RD, and optic neuropathy can cause a significant decrease in vision. A decrease in SBP, DBP, MAP, and SCT correlate significantly with visual outcomes.

## Introduction

Malignant hypertension (MH) is a medical emergency due to the acute rise of systemic blood pressure (BP), resulting in organ damage [1]. The latest guidelines define hypertensive crisis or emergency as systolic BP (SBP) ≥ 180 mmHg and/or diastolic BP (DBP) ≥ 120 mmHg with target organ damage [2,3]. Fundus lesions in MH can present as hypertensive retinopathy, choroidopathy, and optic neuropathy [4]. Spectral-domain optical coherence tomography (SD OCT) provides significantly better image resolution allowing detailed visualization of structural abnormalities in the retina. Morphological findings such as subretinal fluid (SRF), central macular thickness (CMT), and subfoveal choroidal thickness (SCT) can be quantified in OCT and can be used as potential biomarkers in predicting the visual outcome [5,6].

The objective of this study is to correlate visual outcomes in malignant hypertensive retinopathy with changes in systemic causative factors and SD OCT morphologic parameters.

## Materials and methods

This is a prospective observational study conducted between October 2016 to December 2017 in a tertiary care hospital. Patients in the age group 10-40 years presented within two weeks of acute rise of SBP ≥ 180 mmHg and/or DBP ≥ 120 mmHg were included in this study.

Patients having hazy media that obscures good fundus visualization and OCT image acquisition, presence of other associated microvascular diseases such as diabetic retinopathy and retinal vein occlusion, macular pathology, history of recent ocular surgery or trauma, and follow-up less than three months were excluded from this study.

The study conducted adhered to the tenets of the Declaration of Helsinki, and the study protocol was approved by the Institutional Ethics Committee. Patient demographic data, underlying disease, SBP, and DBP at presentation were noted before treatment started. Mean arterial pressure (MAP) was calculated from SBP and DBP measurements.

Ophthalmological examination

At baseline, a detailed ophthalmological examination was done, including best corrected visual acuity (BCVA), slit lamp examination of the anterior segment, intraocular pressure (IOP) measurement, dilated fundus examination with indirect ophthalmoscopy, and fundus photography. SD-OCT images obtained from each eye as per the described protocol. Fundus fluorescein angiography (FFA), indocyanine green angiography (ICG), visual field (VF) analysis, visual evoked potential (VEP) study, and other ancillary tests were conducted when fundus findings were atypical or BCVA did not match with clinical findings.

Imaging protocol

OCT images were acquired using Cirrus HD-OCT MODEL 500 (Carl Zeiss Meditec Jena, Germany). Additionally, 512×128 macular cube scans, five-line raster scans, and enhanced depth imaging (EDI) scans were obtained. Quantitative assessment of CMT, SRF, and SCT was done. A central macular thickness (CMT) was measured automatically via the OCT software. SRF height under the fovea was measured manually on five-line raster scan using the OCT system’s built-in calipers by drawing a perpendicular line between the neurosensory retina and the inner edge of the retinal pigment epithelium (RPE). SCT was measured manually in five-line raster scans with EDI as the linear measurement between the outer border of RPE to the choroidoscleral junction in the center of the fovea. OCT images were also used to evaluate the structural integrity of the retinal layers and any other morphological abnormality of the retinal or choroid.

Patients were followed up monthly for three months to note the pattern of resolution. All the baseline ophthalmological examinations and blood pressure measurements were repeated at three months. Primary outcome measures were visual acuity, hemodynamic parameters (SBP, DBP, MBP), and SD OCT parameters (CMT, SRF, and SCT).

Statistical analysis

Data for continuous variables were expressed as mean ± standard deviation and categorical variables in number (%). BCVA measurements were converted to decimal values for analysis. Paired samples t-test was used to compare the mean value between baseline and final observations of BCVA, SBP, DBP, and MAP with CMT, SRF height, and SCT. A Pearson correlation coefficient was used to calculate the strength of the linear relationship between changes in clinical variables and changes in BCVA. Statistical analyses were performed using the software Statistical Product and Service Solutions (SPSS, version 21; IBM SPSS Statistics for Windows, Armonk, NY). P values < 0.05 were considered statistically significant.

## Results

A total of 33 patients (66 eyes) fulfilled all criteria and were included in the study for analysis. Demographic profile and clinical characteristics are given in Table 1.

**Table 1:** Clinical Characteristics of Patients with Malignant Hypertension

Characteristics	Number of patients (N=33)
Male:female (%)	23:10 (69.7: 30.3)
Age (years)	28.27±8.65 (range 10-40)
Etiology	Renal vascular (16)
	Renal parenchymal (11)
	Pheochromocytoma (4)
	Preeclampsia (2)

**Figure 1:**
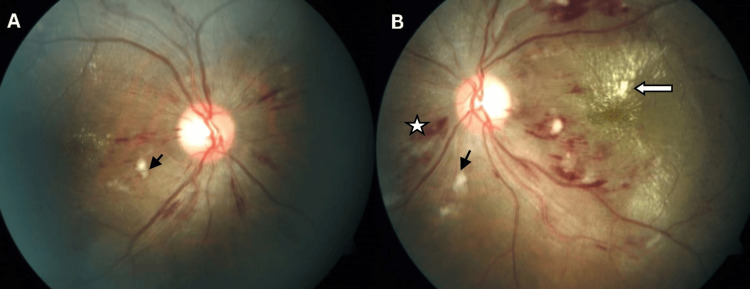
Fundus Picture Fundus picture of a patient with malignant hypertension: Right eye (A) and left eye (B) showing mild disc edema, peripapillary splinter hemorrhages (white asterisk in the left eye), cotton wool spots (black arrows), and hard exudates at the macula (white solid arrow) more marked in the left eye

The most common clinical presentation was the presence of optic disc edema with intraretinal exudates forming complete or incomplete macular stars, peripapillary cotton wool spots, and retinal hemorrhages. Some degree of vascular tortuosity was noted in the majority of patients. There was a bilateral presentation in all cases, but a great degree of asymmetry was noted in a few patients (Figure 1).

**Figure 2:**
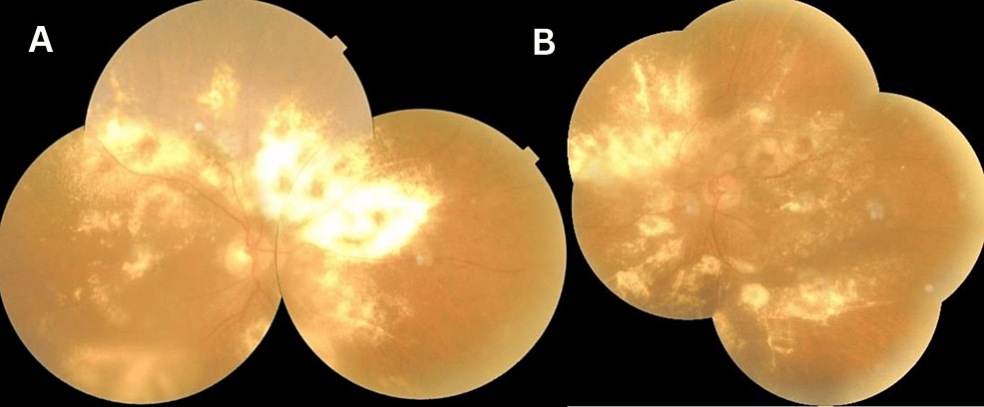
Fundus Picture Montage fundus image of a patient with malignant hypertension: Right eye (A) and left eye (B) showing extensive exudation in all quadrants of both eyes.

Elschnig spots were noted in 10 patients, and exudative retinal detachment (RD) was seen in six patients. Two young patients having pre-eclampsia were presented with pure hypertensive choroidopathy without any signs of retinopathy. In one patient, there was extensive exudation without exudative RD (Figure 2).

The fundus findings are summarized in Table 2.

**Table 2:** Fundus Findings of Malignant Hypertension Patients at Presentation

Fundus findings	Number of eyes (N=66)	Percentage (%)
Optic disc edema	62	94
Tortuosity of blood vessels	62	94
Hard exudates in macula	57	86
Splinter hemorrhage	56	85
Cotton wool spots	53	80
Elschnig spots	10	15
Exudative retinal detachment	6	9
Ischemic Optic neuropathy	3	5
Extensive exudation without retinal detachment	1	2

An SD OCT study shows hyperreflective dots around RPE, basement membrane in the majority of cases. SRF with or without intraretinal fluid was present in 54 eyes. In all cases, SRF and macular edema resolved at three months follow-up after starting antihypertensive medications, but hyperreflective dots took a longer time to resolve. Other morphologic characteristics such as thickening and wrinkling of the nerve fiber layer, disruption of the photoreceptor layer, and clumps of hyperreflective material were noted (Table 3).

**Table 3:** SD OCT Morphologic Characteristics of Malignant Hypertension Patients SD OCT - Spectral Domain Optical Coherence Tomography

SD OCT parameters	Number of eyes (N=66)	Percentage (%)
Hyperreflective dots	63	95
Subretinal fluid	54	82
Intraretinal fluid	48	73
Thickening and wrinkling of the nerve fiber layer	30	46
Clumps of hyperreflective materials	22	33
Loss of Photoreceptor layer integrity	5	8

At the final measurement, mean SBP, DBP, MAP, CMT, SRF, and SCT all decreased significantly from baseline (p<0.05). Improvement in mean BCVA at three months was also statistically significant (p<0.05) (Table 4).

**Table 4:** Change in the Scores Between Baseline and Final Clinical and SD OCT Parameters BL - Baseline, BCVA - Best Corrected Visual Acuity in Decimal, SBP - Systolic Blood Pressure in mmHg, DBP - Diastolic Blood Pressure in mmHg, MAP - Mean Arterial Blood Pressure in mmHg, CMT - Central Macular Thickness in μm, SCT - Subfoveal Choroidal Thickness in μm, SRF - Subretinal Fluid in μm

Variables (N=66)	Pairs	Mean ± SD	T value	P value
BCVA	BL	0.31 ± 0.26	13.53	<0.001
	Final	0.75 ± 0.34		
SBP	BL	211.36 ± 20.93	21.50	<0.001
	Final	159.06 ± 17.40		
DBP	BL	123.58 ± 5.86	50.91	<0.001
	Final	87.15 ± 6.47		
MAP	BL	152.82 ± 6.47	36.39	<0.001
	Final	110.48 ± 9.26		
CMT	BL	424.71± 187.67	8.61	<0.001
	Final	254.44 ± 27.50		
SCT	BL	330.61 ± 51.53	7.51	<0.001
	Final	244.91 ± 19.81		
SRF	BL	138.70 ± 147.44	7.64	<0.001
	Final	0.00 ± 0.00		
Paired samples t-test used to test the mean difference between pre- and post observations. P<0.05 significant. The same hemodynamic parameters measured from each patient were used for both eyes.				

Changes in SBP, DBP, MAP, and SCT were negatively correlated with changes in BCVA (each p<0.001), whereas insignificant poor negative correlations were observed between CMT and SRF to BCVA (each p>0.05). The hemodynamic parameters were observed from the 33 patients, whereas visual acuity and SD OCT parameters were measured for the 66 eyes; that is, each patient had two observations (one for the right eye and another for the left eye). Further same hemodynamic parameters were used for both eyes while estimating the correlation (Table 5).

**Table 5:** Correlation Between Clinical Variables SBP - Systolic Blood Pressure, DBP - Diastolic Blood Pressure, MAP - Mean Arterial Blood Pressure, CMT - Central Macular Thickness, SCT - Subfoveal Choroidal Thickness, SRF - Subretinal Fluid

Changes in clinical variables (N=66)		SBP	DBP	MAP	CMT	SCT	SRF height
Visual acuity	Correlation coefficient (r)	-0.745	-0.549	-0.752	-0.048	-0.416	-0.043
	P value (two sided)	<0.001	<0.001	<0.001	0.704	0.001	0.729
The Pearson correlation coefficient was calculated between the clinical variables. Result indicated that there was significant negative correlation between visual acuity difference and difference of SBP, DBP, MAP, and SCT (n=66, p<0.05). The same hemodynamic parameters measured from each patient were used for both eyes.							

**Figure 3:**
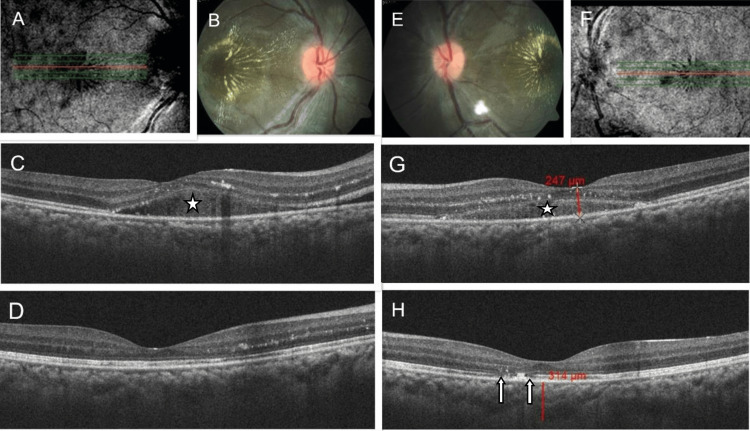
Fundus and SD OCT Macular Scan Image Fundus and SD OCT macular scan image of a patient with malignant hypertension: Infrared image obtained during OCT scan at presentation of the right eye (A) and the left eye (B). Fundus photos of the right eye (B) and the left eye (E) showing optic disc edema, blood vessel tortuosity, hard exudates at the macula in both eyes, and one cotton wool spot inferiorly in the left eye. SD OCT scan of the macula (five-line raster) at presentation of the right eye (C) and the left eye (G) showing hyperreflective dots and subretinal fluid in both eyes (white asterisk). The SD OCT scan of the macula (five-line raster) at three months of the right eye (D) and the left eye (H) shows resolution of subretinal fluid in both eyes, persistent hyperreflective dots more in RE, and photoreceptor layer disruption seen in the left eye (white arrows).

Analyzing the eyes with poor visual recovery showed that macular and choroidal ischemia, exudative RD, and ischemic optic neuropathy were the main factors. Photoreceptor layer disruption was another independent prognostic factor (Figure 3).

## Discussion

In this study, we correlated visual outcomes with changes in systemic causative factors and SD OCT morphologic parameters of the retina and choroid. SD OCT, which provided a near histological section in vivo, helped explore different anatomical changes in the retina and choroid that led to persistent decreased vision despite correction of causative systemic factors. We also studied different ocular presenting signs in malignant hypertension.

The most common clinical presentation appreciable in fundoscopy was a constellation of a number of signs such as optic disc edema, tortuosity of blood vessels with variable amounts of intraretinal exudates forming complete or incomplete macular star, peripapillary cotton wool spots, and retinal hemorrhages. A great degree of asymmetry can be noted between the eyes of the same individual. In our previous articles, we have documented several presentations that can cause diagnostic dilemmas [7,8]. In cases presenting first to an ophthalmologist with atypical pictures such as mild disc edema in the absence of other signs, asymmetry in exudation, presence of only choroidopathy, extensive exudation without other signs of retinopathy or choroidopathy, and disc edema superimposed on chronic retinal disease, malignant hypertension should be excluded first with high clinical suspicion. Early diagnosis will prevent ocular and systemic morbidity as well as mortality [9].

An acute rise in BP damages retinal blood vessels and increases their permeability, resulting in hard exudates and intraretinal fluid. Although Ahn et al. [6] have demonstrated more extensive areas of retinal exudates in patients with more severely elevated BP, we did not notice any such trend. There was a difference in the amount of exudation in the two eyes of the same person although systemic exposure was the same. Similarly, the resolution course was also different in two eyes. Hence, some intraocular factors might also account for this variation.

Hypertensive choroidopathy is mostly seen in younger patients with malignant hypertension because of more flexible blood vessels with contractile function [10,11]. Severely elevated blood pressure results in fibrinoid necrosis of the arterioles and non-perfusion of the choriocapillaris. Resultant ischemia alters retinal pigment epithelium and outer blood-retinal barrier function. It also increases choroidal permeability with an increase in choroidal thickness. Eventually fluid accumulates in subretinal space, causing serous retinal detachment, exudative RD [12-14]. Our study supports the finding of increased choroidal thickness due to malignant hypertension, which decreases with the stabilization of BP.

The most common SD-OCT morphologic finding in our study was hyperreflective dots. They are intraretinal focal transudates that persist for longer periods. We found intraretinal fluid (IRF) and SRF in around two-thirds of cases. At three months follow-up, SRF and IRF were completely resolved in all cases.

Thickening and wrinkling of the nerve fiber layer were seen in half of the patients. They are believed to be due to ischemia, which on resolution can cause nerve fiber layer defects. Irregular reflections in OCT images can also be due to cotton wool spots and flame-shaped retinal hemorrhages. Cotton wool spots represent nerve fiber layer infarct. Although they resolve early within six weeks of control of BP, they are clinically important because an increase in their number indicates significant ischemia.

Previous studies have found increased SCT in hypertensive retinopathy [15,16]. Our study also supports this finding. Significant reduction in retinal and choroidal capillary perfusion is found in hypertensive crisis by optical coherence tomography angiography [17].

With control of BP, there was a rapid resolution of retinal and choroidal changes as evident clinically as well as in SD OCT. At final the measurement, mean SBP, DBP, MAP, CMT, SRF, and SCT all decreased significantly from baseline (p<0.001). Improvement in mean BCVA at three months was also statistically significant (p<0.001). In our study, visual improvement was strongly correlated with changes in SBP, DBP, and MBP. Among SD OCT morphologic features, a change in SCT was strongly correlated with visual improvement. A correlation between changes in CMT, SRF, and visual acuity was not statistically significant. To understand the reason, we analyzed those cases where visual recovery was not complete. Macular and choroidal ischemia, exudative RD, ischemic optic neuropathy, and loss of photoreceptor layer integrity were noted in those cases.

Ahn et al. gave a modified three-step grading system based on fundoscopic findings and OCT images showing the presence or absence of SRF as an indicator of visual prognosis [6]. Whether the presence of SRF is good or bad is currently debatable. Many studies on vascular diseases such as diabetic retinopathy, retinal vein occlusion, and age-related macular degeneration have shown the presence of SRF has no impact on treatment outcome or visual prognosis. Rather some have found favourable outcomes with baseline SRF [18-21]. Although photoreceptor defects can occur following the resolution of chronic SRF in conditions such as chronic central serous chorioretinopathy and long-standing RD [22,23], in malignant hypertension where SRF or IRF respond early to systemic treatment, their impact on visual prognosis is debatable. We also noted that, in some patients, despite comparable fundus picture clinically, as well as SRF amount in the SD OCT measurement in both eyes, photoreceptor layer dysfunctions persisted in one eye only at the final visit (already demonstrated in Figure 3). From these observations, we feel that photoreceptor layer integrity can be an independent prognostic factor unrelated to SRF. The effect of local intraocular factors also can not be ruled out.

The strength of our study is that it is a prospective study with uniform follow-up of all patients. The limitation of our study is that the follow-up period is short. Although the majority of clinical signs resolve quickly with control of BP, it is desirable to see long-term effects.

## Conclusions

Malignant hypertension can present with diverse posterior segment manifestations in the eyes. Clinical signs, although bilateral in the majority of cases, can be highly asymmetric between eyes. Recognizing these signs along with high clinical suspicion of this entity will prevent unnecessary investigations and delays in treatment.

In malignant hypertensive retinopathy, SRF with macular edema is the major cause of mild-to-moderate decrease BCVA at presentation, but profound loss of vision is caused by macular ischemia, exudative RD, and optic neuropathy. Incomplete visual recovery after resolution of macular edema and SRF was due to disruption of the photoreceptor layer, the persistence of clumps of hard exudates, and macular thinning, as evident in SD OCT. A decrease in SBP, DBP, MAP, and SCT correlated significantly with changes in BCVA, but CMT and SRF did not correlate significantly with visual outcomes. Further studies involving larger sample sizes, multimodal investigations, and longer follow-ups are needed to confirm our findings.
